# Supplementary material for: A Theoretical Model for the Associative Nature of Conference Participation
Source: PLoS One. 2016 Feb 9;11(2):e0148528. doi: 10.1371/journal.pone.0148528 (PMC4747514; doi:10.1371/journal.pone.0148528)
Supplement: S1 File — Proportion of conference participants g with x conference attendances who are going to attend the conference next year (Figure A). Proportion of conference participants ρ with n missed conferences after x-th conference attendance who are going to skip the conference next year, but will take part at some future conference from the observation period (Figure B). Pages on the web from which we downloaded conference data (Table A). Summary of the conference data. Columns 2 and 3 indicate for each conference the year in which data we have collected begin (Y0) and end (Yf). The total number of different participants at the conference during that period of time is given in column 4 (Table B). The number of participants at the conference per year (Table C). Log likelihood ratio R and the π-value compare the fit to the power-law with the fit to the power-law with an exponential cutoff for the probability distribution of number of conferences at which each author appears (Table D). Log likelihood ratio R and the π-value compare the fit to the power-law with the fit to the power-law with an exponential cutoff for the probability distribution of the number of successive participations at the conference (Table E). Log likelihood ratio R and the π-value compare the fit to the exponential with the fit to the power-law with an exponential cutoff for the probability distribution of the time lag between two consecutive conference participations (Table F). The optimal parameter values of the model for each conference (Table G). Stagnancy rate 1 − g(1, 0) at t = 1 for each conference and exponent α of power-law with an exponential cutoff distribution fit with the corresponding conference order (Table H). (PDF) [file pone.0148528.s001.pdf]

Supplementary Information: A theoretical model for the associative nature of conference participation

Jelena Smiljanić[1], Arnab Chatterjee[3], Tomi Kauppinen[4], and Marija Mitrović Dankulov[1,*]

[1]Scientific Computing Laboratory, Institute of Physics Belgrade, University of Belgrade, Pregrevica 118, 11080 Belgrade, Serbia
[2]School of Electrical Engineering, University of Belgrade, P.O. Box 35-54, 11120 Belgrade, Serbia
[3]Condensed Matter Physics Division, Saha Institute of Nuclear Physics, 1/AF Bidhannagar, Kolkata 700064, India
[4]Aalto University School of Science, P.O. Box 11000. FI-00076 AALTO, Finland
[*]mitrovic@ipb.ac.rs

January 23, 2016

1 Data

1.1 Conference description

The American Physical Society March Meeting (APSMM) is the world's largest condensed matter physics conference with more than 70 years history. It is organized annually at various locations in The United States. The conference attracts researchers from research institutions, universities, and industry from all around the world.

The APS April Meeting (APSAM) conference is dedicated to the topics from the astrophysics, gravitational physics, nuclear physics, and particle physics. Likewise March Meeting, the conference takes place at various locations in The United States each year.

The Annual Meeting of the Society for Industrial and Applied Mathematics (SIAM) has been held since 1984 at various locations in The North America. Topics covered at the SIAM conferences include applied and computational mathematics and applications.

The Neural Information Processing Systems (NIPS) Conference has been held since 1988 at various locations in The United States, Canada and Spain. Neural information processing intends to emerge fields such as machine learning, statistics, applied mathematics and physics. The acceptance rate is about 50%.

The aim of The International Conference on Supercomputing (ICS) is to promote an international forum for the presentation and discussion on the various aspects of high-performance computing systems. The ICS conference has been sponsored by The Association for Computing Machinery (ACM). The conference is organized annually since 1988 at various locations. The overall acceptance rate is 26%.

The Annual International Conference on Research in Computational Molecular Biology (RECOMB) has been held since 1997 at various locations. At RECOMB emphasis is placed on connecting the biological, computational, and statistical sciences. The overall acceptance rate is 20%.

The list of links to the conference data and proceedings is given in Table A, while the Table C lists the sizes of all six conferences for all years covered in the data set. The number of participants is calculated after the name disambiguation.

1.2 Data description

Conference	Link to the conference data set
APSMM	http://www.aps.org/meetings/baps/
APSAM	http://www.aps.org/meetings/baps/
SIAM	http://www.siam.org/meetings/archives.php#AN
NIPS	http://papers.nips.cc/
ICS	http://dl.acm.org/event.cfm?id=RE215&tab=pubs
RECOMB	http://www.recomb.org/history

Table A: Pages on the web from which we downloaded conference data.

Conference	Y_0	Y_f	*Number of participants*
APSMM	1994	2014	78544
APSAM *	1994	2014	16264
SIAM **	1994	2014	8879
NIPS	1988	2014	6902
ICS	1988	2014	2504
RECOMB	1997	2014	1564

* Data are not available for 1999.
** Data are not available for 2007 and 2011.

Table B: Summary of the conference data. Columns 2 and 3 indicate for each conference the year in which data we have collected begin (Y_0) and end (Y_f). The total number of different participants at the conference during that period of time is given in column 4.

	APSMM	APSAM	SIAM	NIPS	ICS	RECOMB
1988	-	-	-	214	132	-
1989	-	-	-	205	121	-
1990	-	-	-	297	123	-
1991	-	-	-	302	116	-
1992	-	-	-	270	112	-
1993	-	-	-	301	114	-
1994	9660	3309	540	270	114	-
1995	9897	1947	425	292	144	-
1996	9991	2356	279	289	127	-
1997	9191	3388	579	289	109	111
1998	10924	2301	456	298	158	120
1999	20426	-	367	296	172	121
2000	10816	1744	403	307	105	150
2001	12401	1818	823	396	146	101
2002	11944	2446	1115	432	118	98
2003	13548	2127	642	469	103	95
2004	14595	1668	767	492	102	136
2005	14673	1140	792	515	165	141
2006	16484	1008	945	479	124	154
2007	16655	943	-	530	96	123
2008	16441	1473	1053	633	132	142
2009	16775	1630	1054	654	242	127
2010	17790	1342	1166	733	127	157
2011	18368	1088	-	746	171	167
2012	22343	1480	1223	938	133	148
2013	21510	1430	1205	884	210	125
2014	22789	1704	1407	1064	147	137

Table C: The number of participants at the conference per year.

2 Functional fits

We use maximum-likelihood to estimate the parameters of three different functions, exponential, power-law and power-law with an exponential cutoff for the distributions of total number of participations, the number of and the time lag between two successive participations. Further on, we calculate the log-likelihood ratio, $\mathcal{R}$, and π-value [1] between different fits in order to estimate which of the three different functional forms the best fits with the empirical observations. The Tables D and E show $\mathcal{R}$, and π-value calculated for the comparison between truncated power-law and pure power-law for total and successive number of participations, while Table F shows the comparison between fits of exponential and truncated power-law to the distribution of time lags. These results and visual inspection show that the power-law with an exponential cutoff is the best fit for all three empirical distributions, and for all six conferences.

	$\mathcal{R}$	π
APSMM	-1758.44	0.0
APSAM	-36.89	0.0
SIAM	-75.26	0.0
NIPS	-76.64	0.0
ICS	-8.54	3.60e-05
RECOMB	-7.22	1.45e-04

Table D: Log likelihood ratio $\mathcal{R}$ and the π-value compare the fit to the power-law with the fit to the power-law with an exponential cutoff for the probability distribution of number of conferences at which each author appears.

	$\mathcal{R}$	π
APSMM	-554.05	0.0
APSAM	-0.77	0.21
SIAM	-17.98	2.01e-09
NIPS	-17.52	3.24e-09
ICS	-4.99	1.57e-03
RECOMB	-1.48	0.09

Table E: Log likelihood ratio $\mathcal{R}$ and the π-value compare the fit to the power-law with the fit to the power-law with an exponential cutoff for the probability distribution of the number of successive participations at the conference.

	$\mathcal{R}$	π
APSMM	-756.91	0.0
APSAM	-34.59	1.11e-16
SIAM	-11.54	1.55e-06
NIPS	-58.22	0.0
ICS	-7.64	9.24e-05
RECOMB	-3.60	7.27e-03

Table F: Log likelihood ratio $\mathcal{R}$ and the π-value compare the fit to the exponential with the fit to the power-law with an exponential cutoff for the probability distribution of the time lag between two consecutive conference participations.

3 Model of conference attendance dynamics

3.1 Participation probability

Figure A shows how the probability to attend the next meeting is changing with the number of previous attendances, calculated from the empirical data. We see that for all six conferences this probability grows for a small number of attendances. The saturation or decrease in the probability for a large number of previous participations, observed for some conferences, occurs due to a small number of observations for the large number of participations/length of pauses.

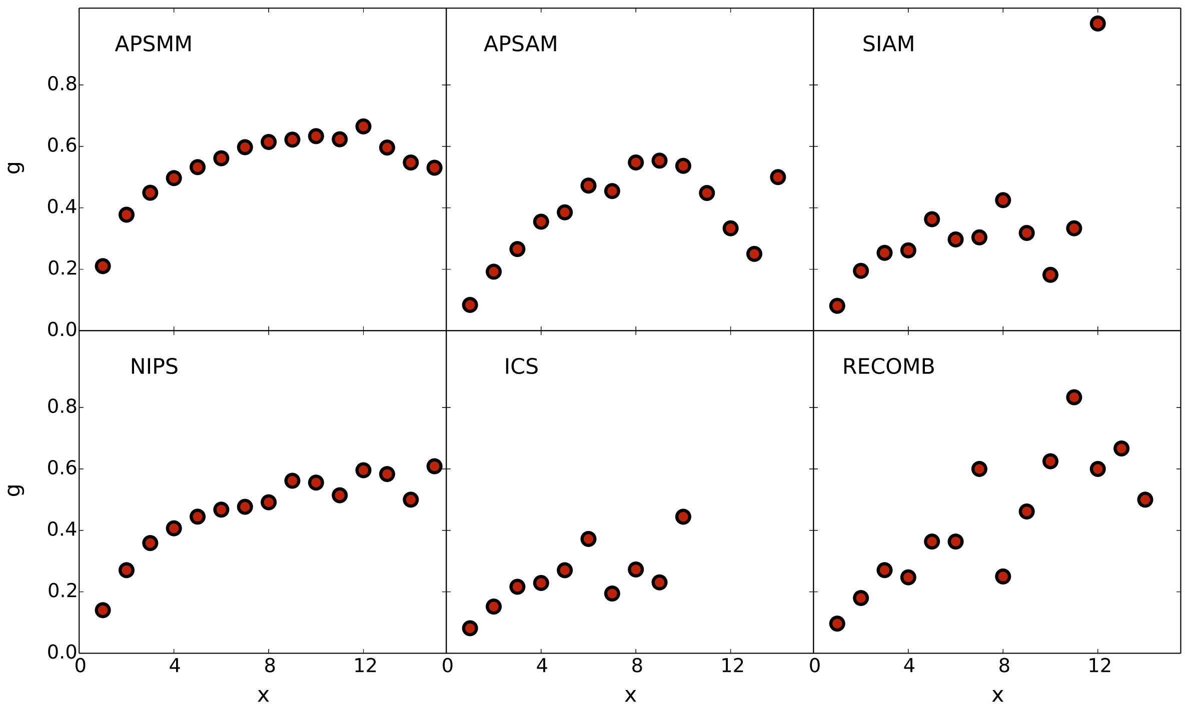

Figure A: Proportion of conference participants g with x conference attendances who are going to attend the conference next year.

Figure B shows how the probability to not attend the next meeting, $\rho = 1 - g$, increases with the number of non-participations, n, for the fixed number of previous participations x. We see that this probability is higher for smaller x and the same value of n.

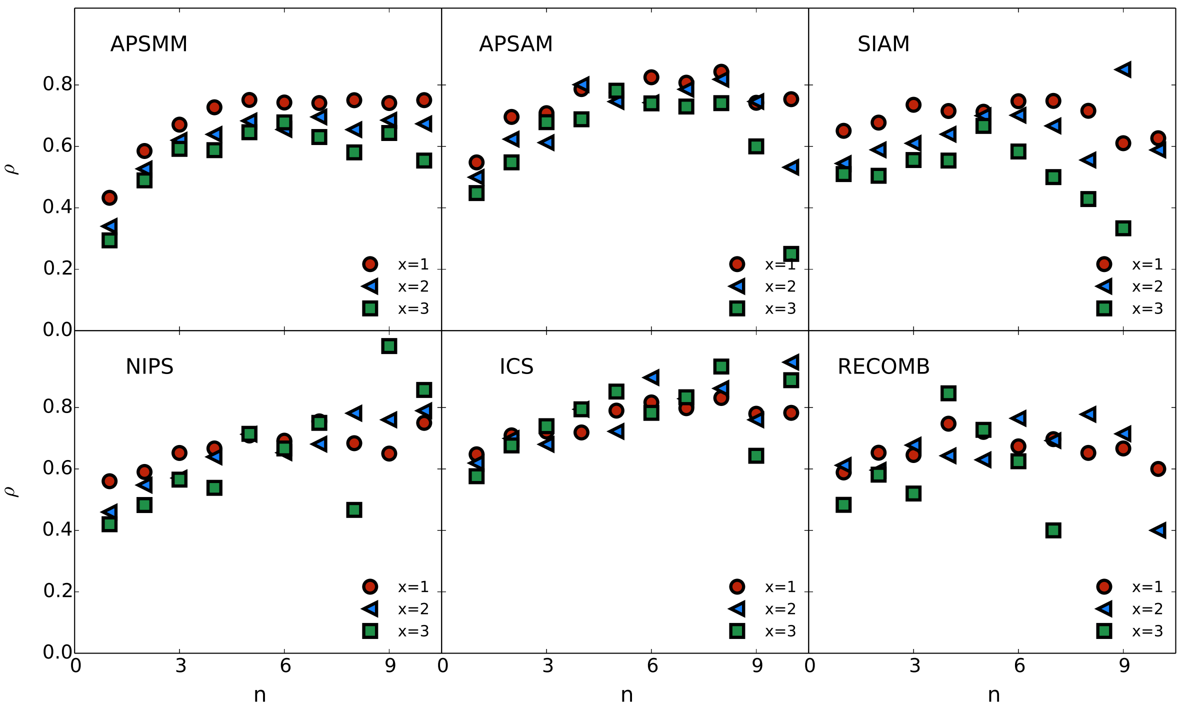

Figure B: Proportion of conference participants ρ with n missed conferences after x-th conference attendance who are going to skip the conference next year, but will take part at some future conference from the observation period.

3.2 Parameter estimation

The Table G shows the optimal parameter values of the model for the six different conferences. In Table H we show the estimated values of conference inclusiveness, $g(1, 0)$, and the order of the conferences according to this value and the value of exponent α.

	y_0	H	p
APSMM	2	0.165	1.550
APSAM	4	0.135	1.700
SIAM	4	0.155	1.525
NIPS	3	0.130	1.525
ICS	4	0.135	1.575
RECOMB	3	0.175	1.675

Table G: The optimal parameter values of the model for each conference.

	order	$1 - g(1, 0)$	order	α
APSMM	1	0.2546	1	1.64
APSAM	6	0.0865	6	2.62
SIAM	4	0.1077	3	2.10
NIPS	2	0.1577	2	1.93
ICS	5	0.1012	5	2.51
RECOMB	3	0.137	4	2.31

Table H: Stagnancy rate $1 - g(1, 0)$ at $t = 1$ for each conference and exponent α of power-law with an exponential cutoff distribution fit with the corresponding conference order.

3.3 Iterative method

The model evolution equations cannot be solved analytically, thus we use a numerical simulation and an iterative method. Here we explain the iterative method in details. Figure C is a schematic representation of the evolution process, which is a type of a Markovian process between states. Each state represents the number of participations. At each time step, the scientist can either attend a conference, with probability $g(x, t - x)$, and move one state right and increase the total number of participations, or not, and thus stay at the same state.

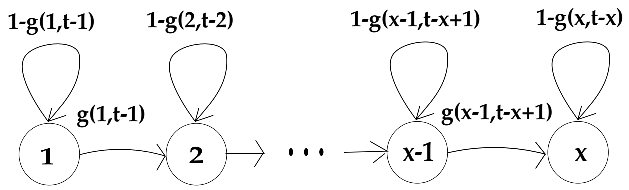

Figure C: Scheme of the evolution of the process through one time step.

To mathematically describe this evolution process we construct the transition probability matrices $M(t)$ of sizes $t \times t$, with elements

$$M_{i,j}(t) = \begin{cases} 1 - g(i, (t-1) - i), & j = i \text{ and } i < t, \\ g(i, (t-1) - i), & j = i + 1 \text{ and } i < t, \\ 0, & \text{otherwise.} \end{cases}$$

for $t \geq 2$ and $M(t = 1) = \mathrm{I}$ at $t = 1$. The diagonal elements $M_{i,i}(t)$ define the probability that a participant who has i attendances on $t - 1$ conferences, does not attend conference at time t, while $M_{i,i+1}(t)$ represents the probability for the transition $i \to i + 1$. We assume that the termination time of a conference career T is the same for all participants and observe matrix

$$\mathcal{M} = M'(1)M'(2) \dots M'(T-1)M(T) \tag{1}$$

where $M'(t)$ is the matrix $M(t)$ expanded to the size $T \times T$ by adding $T - t$ zero rows and columns. The resulting matrix $\mathcal{M}$ has non-zero elements at the first row, and other elements are 0. Each element $\mathcal{M}_{1,i}$ of the matrix $\mathcal{M}$ is the sum of all the possible combinations of attended and skipped conferences that result in i total participations at time T. Otherwise stated, the ratio of authors who attended i conferences is given by $\mathcal{M}_{1,i}$.

Based on this consideration, we next examine the probability distribution of the total number of participations when the termination of attendance occurs at random with some constant probability H. We generate matrices $\mathcal{M}(t)$:

- $t = 1$, $\mathcal{M}(1) = HM(1)$;
- $t = 2$, $\mathcal{M}(2) = \left[\frac{1-H}{H} M'(1)\right] [HM(2)]$;
- $t = 3$, $\mathcal{M}(3) = \left[\frac{1-H}{H} \mathcal{M}'(2)\right] [HM(3)]$;
- $t = T_{max}$, $\mathcal{M}(T_{max}) = \left[\frac{1-H}{H} \mathcal{M}'(T_{max} - 1)\right] [HM(T_{max})]$;

where $\mathcal{M}'(t)$ is the matrix $\mathcal{M}(t)$ expanded to the size $t+1 \times t+1$ by adding a zero row and column. Each of elements $\mathcal{M}_{1,i}(t)$ of the matrix $\mathcal{M}(t)$ gives a ratio of participants that terminated their conference career at time t with i participations. We can choose T_{max} to stop the calculation when $\sum_{i=1}^{T_max} \mathcal{M}_{1,i}(T_max) \to 0$. Then, probability distribution of the total number of participations $P(x)$ is obtained by summing over all possible career termination times

$$P(x) = \sum_{t=1}^{T_{max}} \mathcal{M}_{1,x}(t). \tag{2}$$

References

[1] A. Clauset, C. R. Shalizi, and M. E. J. Newman, "Power-law distributions in empirical data," *SIAM Review*, vol. 51, no. 4, pp. 661–703, 2009.
